# Disclosing an autism diagnosis improves ratings of candidate performance in employment interviews

**DOI:** 10.1177/13623613231203739

**Published:** 2023-10-26

**Authors:** Jade Eloise Norris, Rachel Prosser, Anna Remington, Laura Crane, Katie Maras

**Affiliations:** 1 University of Bristol, UK; 2 Oxford Health NHS Foundation Trust, University of Oxford, UK; 3 University College London, UK; 4 University of Bath, UK

**Keywords:** adults, autism spectrum disorders, cognition (attention, learning, memory), communication and language, policy, professional development, quality of life, vocational/labour force participation

## Abstract

Employment interviews can be challenging for autistic people. In addition to standard interview procedures often being inaccessible, features associated with autism (including atypical emotional expression, eye-contact, and gestures) may lead to negative first impressions. Research using vignettes and ‘thin slices’ of communication indicate that perceptions of an autistic individual can be improved when their autism diagnosis is disclosed. Here, we examined the impact of diagnostic disclosure on judgements of autistic adults undergoing an entire mock employment interview. A total of 119 participants (known as ‘raters’) watched a video of an autistic candidate’s mock employment interview, after which they provided overall impressions of the candidate on factors such as confidence, motivation, and knowledgeability. Raters were either (1) unaware of the interviewee’s diagnosis, (2) aware of their diagnosis, or (3) aware of their diagnosis and provided with information about autism. Results indicate that diagnostic disclosure improved perceptions of autistic candidates across all dimensions compared to when raters were unaware of their diagnosis. The provision of additional information about the diagnosis did not further improve ratings. The findings have important implications for employers and autistic people, who should consider the potential impact of diagnostic disclosure prior to interviewing for a role.

**Lay Abstract:**

Employment interviews are challenging for many autistic people, for example, due to difficulties with interpreting questions. Autistic people also have differences in non-verbal communication, such as emotional expression, eye-contact, and gestures, with research showing that these factors negatively affect first impressions. Some studies have shown that perceptions of autistic people are more positive when the person observing them, such as an interviewer, is already aware of their diagnosis. However, previous research has not studied how disclosing one’s autism diagnosis affects perceptions of a candidate undergoing a full employment interview. Participants in this study acted as raters, who watched a video of an autistic person undergoing a mock employment interview with a researcher, and then rated their overall impressions of them on factors important to real-world hiring decisions, such as confidence, motivation, and knowledgeability. Prior to watching the interview, raters were either (1) *not* aware of the interviewee’s diagnosis, (2) made aware of their diagnosis, or (3) made aware of their diagnosis *and* provided with additional information about autism, such as differences in behaviours and communication. The results show that disclosing an autism diagnosis improved ratings compared to not disclosing the diagnosis. Providing additional information about autism alongside the diagnostic label did not improve ratings further. The findings are important for employers and autistic people; employers should consider improving procedures by which autistic people can disclose their diagnosis prior to interview should they wish, and autistic people may wish to consider the potential benefits of disclosing their diagnosis.

## Introduction

Gaining meaningful employment presents a significant challenge for many autistic people, and autistic adults in the United Kingdom are the most underemployed disability group (Office for National Statistics, 2021). Navigating employment interview questions is known to be a key challenge for autistic adults (Maras et al., 2020), due to factors such as difficulties in understanding interviewers’ implicit expectations, plus relational memory and executive function differences (which become particularly problematic under the ubiquitous use of open-ended questioning; see, for example, Maras et al., 2020; Norris et al., 2020). In addition, behavioural characteristics associated with autism such as atypical emotional expression, eye-contact, and gestures are all factors implicated in negative first impressions of autistic people (Sasson & Morrison, 2019).

Judgements about autistic people can be improved when their diagnosis is disclosed (Brosnan & Mills, 2016; Crane et al., 2018; Maras et al., 2018; Sasson & Morrison, 2019). Providing a diagnostic label, alongside information about autism, provides a reason for atypical behaviour, allowing an observer to apply an alternative explanation for unexpected behaviours (e.g. that atypical eye contact during an interview is linked to autistic differences, as opposed to disinterest in the role; Brosnan & Mills, 2016; c.f. Kelley, 1973).

Literature has begun to emerge on autism diagnostic disclosure in the workplace. For example, a recent study asked lay raters to evaluate ‘thin slices’ of a non-autistic and an autistic actor’s performance in a mock employment interview (Flower et al., 2021). Diagnostic label disclosure improved perceptions, even when the non-autistic interviewee ‘disclosed’ that they were autistic. In addition, although providing more information about autism alongside the diagnosis did not significantly improve ratings further, it did slightly reduce the likelihood of a rater hypothetically deciding not to hire the autistic candidate, indicating that the provision of further information about autism may be important (Flower et al., 2021). However, a limitation of Flower et al.’s (2021) work is that raters were not experienced in employment interviewing, so the findings may not reflect real-world practices. In addition, as recent studies have used limited interview stimuli such as vignette stories and short 10-s clips (Flower et al., 2021; McMahon et al., 2020), it is currently unclear whether diagnosis disclosure (with and without the provision of information about autism alongside the diagnostic label) has a similar effect when a rater is observing a video of an entire interview.

Although such methods have been used to investigate the impact of autism diagnosis disclosure within other contexts, including the Criminal Justice System (e.g. Maras et al., 2018), to our knowledge, no study to date has compared observer (with employment interviewing experience) perceptions of real-time videos of autistic candidates undergoing employment interviews when raters were (1) unaware of their diagnosis, (2) aware of their diagnosis, or (3) aware *and* provided with information about the diagnosis. It was predicted that informing raters of an interviewee’s autism diagnosis would improve their perceptions compared to no disclosure, and that the provision of additional information about autism alongside the diagnostic label may further improve perceptions compared to when raters receive the label alone.

## Method

### Design

This study investigated the impact of diagnostic disclosure (between-subjects): (1) no label, versus (2) label only, versus (3) label plus information, on evaluations of autistic interviewees undergoing mock employment interviews. The dependent variable was quantitative scale ratings of the candidates, measured on nine aspects of performance (see Procedure).

### Participants

Data for the no label condition came from a prior study assessing perceptions of autistic and non-autistic candidates undergoing interviews with standard versus adapted questioning, and rated based on transcripts compared to videos (Norris et al., under review). There were three rater participants for 10 of the videos, and two rater participants for 4 of the videos. Rater participants rated one video each. Participation took ~20 min, and participants were reimbursed £5. All participants watched and rated the video online, with most taking part remotely (*N* = 26), and 12 participating in the laboratory at the University of Bath or University College London.

For the label only and label plus information conditions, an additional 98 raters were recruited, completing the study online (label only = 48; label plus information = 50). For the label only condition, there were three rater participants for 10 of the videos, and two rater participants for 4 of the videos. For the label plus information condition, there were three rater participants for 13 of the videos, and one rater participant for one of the videos. Participation took around 30 min, and participants were reimbursed £8. Three mock-candidates’ videos were not used in this study as they had disclosed their diagnosis during the original interviews. Therefore, data from rater participants in this study which had been collected for these videos (in the label only and label plus information conditions) were excluded from the analyses, such that the final number of rater participants for each condition were: no label *N* = 38; label only *N* = 40; label plus information *N* = 41 (see Table 1).

**Table 1.** Rater participant information.^a^

	No label (*N* = 38)	Label only (*N* = 40)	Label plus info (*N* = 41)
Gender	24 female, 14 male	28 female, 12 male	31 female, 9 male (1 no response)
Age (years)	*M* = 42.50, (*SD* = 15.95), range = 20–71	*M* = 44.40, (*SD* = 10.51), range = 26–64	*M* = 45.17, (*SD* = 10.94), range = 30–67
Interviewing experience (0–6 scale rating)	*M* = 3.50 (*SD* = 1.87), range = 0–6	*M* = 3.60 (*SD* = 1.66), range = 1–6	*M* = 3.63 (*SD* = 1.50), range = 1–6
Autism Awareness Scale (Gillespie-Lynch et al., 2015)^b, c^	*M* = 12.16, *SD* = 4.20, range = 2–21	*M* = 12.88, *SD* = 4.94, range = 2–22	*M* = 13.66, *SD* = 4.71, range = 2–21
Level of Contact Scale (Gardiner & Iarocci, 2014; Morrison et al., 2019)^d^	*M* = 6.05, *SD* = 2.96, range = 1–12	*M* = 6.98, *SD* = 2.74, range = 2–11	*M* = 7.00, *SD* = 3.16, range = 1–11
Social Distance Scale (Gillespie-Lynch et al., 2015)^e^	*M* = 10.16, *SD* = 3.51, range = 6–18	*M* = 9.00, *SD* = 3.36, range = 6–20	*M* = 8.49, *SD* = 2.64, range = 6–15

a One-way ANOVAs indicated that there were no group differences for age (*p* = 0.631), level of interviewing experience (*p* = 0.935), Autism Awareness (*p* = 0.358), Level of Contact (*p* = 0.279), or Social Distance scales (*p* = 0.063).

b We removed item 13; ‘People with autism have empathy’ due to debate regarding this issue (e.g. Milton, 2012).

c Participants responded using a 5-point scale from strongly disagree (−2) to strongly agree (2) on 12 items. Seven items were reverse-scored. Scores were summed to generate a total autism knowledge score that could range from −24 to 24, with a higher score indicating greater levels of autism knowledge.

d Participants read 12 statements describing a relationship/experience/s with autistic people, ranging in intensity/closeness of the relationship from 1 ( ‘I have never observed a person that I was aware was autistic’) to 12 ( ‘I am autistic’). Participants were encouraged to choose as many items as were applicable to them, and their final score was their highest level of contact selected.

e Participants chose a response on a 4-point scale from definitely unwilling (4) to definitely willing (1) (i.e. from least stigma to most stigma). Scores across the six items were summed to generate a total stigma score that ranged from 6 to 24, with a higher score indicating greater stigma.

Across all conditions, raters were recruited on the basis of having some prior experience interviewing people in an employment context, and being unlikely to know/guess that this study was related to autism research (e.g. recruiting via a proxy/researcher who was not primarily linked to an autism research centre). Participants spoke English fluently, had normal/corrected-to-normal vision/hearing, and (to protect stimuli participant) did not work/study at the University of Bath or University College London. All participants also completed brief scales measuring autism knowledge, experience, and stigma after providing their ratings (Table 1).

### Materials

Interview videos were obtained in a previous study investigating employer ratings of autistic and non-autistic candidates during unadapted and adapted interviews (see full procedure in Maras et al., 2020), with videos of 14 autistic candidates undergoing unadapted interviews providing the stimuli for this study.

### Procedure

Participants in the no label condition received no information about the interviewee’s autism diagnosis. Participants in the label only condition were told ‘The person being interviewed has a diagnosis of autism’. Finally, participants in the label plus information condition were provided with the label, as well as brief information about autism, including differences in verbal, non-verbal, and para-verbal behaviours and communication (adapted from Crane et al., 2018; see Supplementary Materials 1). Participants then watched one video of a mock interview, and rated their overall impressions of the candidate on a 5-point scale ranging from ‘not at all’ to ‘extremely’ on; confidence, motivation, knowledgeability, conscientiousness, competence, intelligence, communication skills, likeability, and ease to work with (Huffcutt, 2011).

### Community involvement

There was no community involvement in the reported study.

## Results

To investigate the impact of diagnostic disclosure on impressions of autistic candidates, a repeated measures analysis of variance (ANOVA) was conducted, with disclosure (no label vs label only vs label plus information) as a between-subjects factor, and mean impression ratings as dependent variables (see Table 2).

**Table 2.** Descriptive and inferential statistics for mean interviewee ratings across disclosure conditions.

Rating	No label	Label only	Label plus information	Univariate tests	Within-subjects contrasts – no label vs label
Confident	M = 1.54 (*SD* = 0.80), range = 0.50–3.00	*M* *=* 2.98 (*SD* *=* 0.65), *range* *=* 2.00–4.33	*M* *=* 3.14 (*SD* *=* 0.60), *range* *=* 2.00–4.00	*F*(1.87) = 50.99, *p* < 0.001, η^2^_p_ = 0.80	*F*(1, 13) = 60.06, *p* < 0.001, η^2^_p_ = 0.82
Motivated	*M* *=* 1.80 (*SD* *=* 0.50), *range* *=* 1.33–3.00	*M* *=* 3.27 (*SD* *=* 0.51), *range* *=* 2.33–4.00	*M* *=* 3.17 (*SD* *=* 0.72), *range* *=* 1.67–4.00	*F*(1.61) = 43.56, *p* < 0.001, η^2^_p_ = 0.77	*F*(1, 13) = 62.06, *p* < 0.001, η^2^_p_ = 0.83
Knowledgeable	*M* *=* 1.95 (*SD* *=* 0.63), *range* *=* 1.00–3.00	*M* *=* 3.29 (*SD* *=* 0.46), *range* *=* 2.50–4.00	*M* *=* 3.27 (*SD* *=* 0.57), *range* *=* 2.00–4.33	*F*(1.65) = 32.99, *p* < 0.001, η^2^_p_ = 0.72	*F*(1, 13) = 63.05, *p* < 0.001, η^2^_p_ = 0.83
Conscientious	*M* *=* 2.09 (*SD* *=* 0.58), *range* *=* 1.33–3.33	*M* *=* 3.57 (*SD* *=* 0.38), *range* 3.00–4.33	*M* *=* 3.50 (*SD* *=* 0.81), *range* *=* 2.00–4.67	*F*(1.60) = 23.91, *p* < 0.001, η^2^_p_ = 0.65	*F*(1, 13) = 74.51, *p* < 0.001, η^2^_p_ = 0.85
Competent	*M* *=* 1.85 (*SD* *=* 0.62), *range* *=* 0.67–2.67	*M* *=* 3.12 (*SD* *=* 0.40), *range* *=* 2.67–4.00	*M* *=* 3.20 (*SD* *=* 0.60), *range* *=* 1.50–4.00	*F*(1.93) = 37.30, *p* < 0.001, η^2^_p_ = 0.74	*F*(1, 13) = 43.90, *p* < 0.001, η^2^_p_ = 0.77
Intelligent	*M* *=* 2.10 (*SD* *=* 0.47), *range* *=* 1.00–2.67	*M* *=* 3.41 (*SD* *=* 0.50), *range* *=* 2.67–4.33	*M* *=* 3.45 (*SD* *=* 0.61), *range* *=* 2.00–4.33	*F*(1.65) = 55.08, *p* < 0.001, η^2^_p_ = 0.81	*F*(1, 13) = 145.62, *p* < 0.001, η^2^_p_ = 0.92
Good at communicating	*M* *=* 1.40 (*SD* *=* 0.88), *range* *=* 0.00–3.00	*M* *=* 3.00 (*SD* *=* 0.60), *range* *=* 2.00–4.00	*M* *=* 2.82 (*SD* *=* 0.71), *range* *=* 1.50–4.00	*F*(1.41) = 37.61, *p* < 0.001, η^2^_p_ = 0.74	*F*(1, 13) = 52.64, *p* < 0.001, η^2^p = 0.80
Likeable	*M* *=* 1.82 (*SD* *=* 0.57), *range* *=* 0.67–2.67	*M* *=* 3.33 (*SD* *=* 0.65), *range* *=* 1.67 = 4.00	*M* *=* 3.29 (*SD* *=* 0.84), *range* *=* 1.67–4.33	*F*(1.86) = 58.12, *p* < 0.001, η^2^_p_ = 0.82	*F*(1, 13) = 108.98, *p* < 0.001, η^2^_p_ = 0.89
Easy to work with	*M* *=* 1.68, (*SD* *=* 0.78), *range* *=* 0.00–2.67	*M* *=* 2.86 (*SD* *=* 0.61), *range* *=* 1.67–3.67	*M* *=* 2.79 (*SD* *=* 0.69), *range* *=* 1.67–3.67	*F*(1.84) = 35.73, *p* < 0.001, η^2^_p_ = 0.73	*F*(1, 13) = 51.14, *p* < 0.001, η^2^_p_ = 0.80

Where the assumption of sphericity was violated, Greenhouse–Geisser corrections were applied. Univariate tests indicated significant differences between conditions for all measures, with within-subjects contrasts indicating higher ratings when raters were informed of the interviewee’s autism diagnosis (label only condition) compared to when not informed (no label; *p* < 0.001). The provision of a label plus information did not further improve ratings compared to label only (*p* > 0.05).

## Discussion

In this study, we examined differences between rater perceptions of autistic candidates undergoing mock employment interviews based on level of diagnostic disclosure (e.g. when receiving either no diagnostic information, the candidate’s diagnostic label only, or the label plus information about autism). Results demonstrated that candidates were perceived more favourably when raters were provided with their diagnostic label prior to watching the video, compared to no label (as in Flower et al., 2021; McMahon et al., 2020).

Providing further information about the diagnosis did not additionally improve perceptions over and above provision of the label alone (as in Flower et al., 2021). Previous research from contexts such as the Criminal Justice System found that the provision of information about a person’s autism diagnosis improved mock-juror perceptions of witnesses (Maras et al., 2019), although in the study by Maras et al. (2019) no disclosure was being compared with disclosure plus autism information (i.e. there was no label-only condition). The current findings tentatively suggest that the disclosure of an autism diagnosis alone may have been enough to improve ratings in previous research. Although a caveat to this suggestion is that in this study we recruited raters with employment interviewing experience who, as a result, may have higher levels of experience, knowledge, or education enabling them to apply knowledge about the diagnosis alone. General population-based samples (i.e. representative of jurors) may require additional information about autism. However, it is worth noting that our sample demonstrated similar scores for autism knowledge, experience, and stigma to those in prior research (e.g. Morrison et al., 2019; Sasson & Morrison, 2019).

Importantly moreover, the additional information about autism provided in this study was generic and not tailored to each individual candidate, which may have limited its utility to raters (and thus having less impact on ratings). Indeed, previous findings from Crane et al. (2018) demonstrate mixed findings, with the provision of the diagnostic label alongside autism information improving credibility judgements of one child witness, but not for another child who had more observable autistic behaviours. Crane et al. (2018) concluded that, when engaging in diagnostic disclosure, rather than using generic information about autism, observers should instead be presented with information tailored to the individual (i.e. autistic candidate), outlining the individual’s autism features, and how these may impact upon their performance and behaviours at interview (Crane et al., 2018).

In addition, the perceived importance of the context is likely to impact upon ratings; for example, providing one’s perceptions of a mock employment candidate has different implications from engaging in real-world hiring decisions. It is not yet clear whether the provision of a diagnostic label without further information about its implications could potentially lead to stigmatising effects in other contexts (McMahon et al., 2020). The question also remains as to whether the benefits of diagnostic disclosure shown here are transferable to other employment-related and other contexts, including for example colleague acceptance and understanding.

While the focus in terms of supporting autistic people to gain employment has historically been on training autistic people to answer interview questions in an ‘appropriate’ manner, this study adds to recent findings highlighting that diagnostic disclosure may improve perceptions of candidates and employees (Flower et al., 2021; McMahon et al., 2020; Norris et al., under review). Employers should consider improving the methods by which autistic candidates can disclose their diagnosis prior to interview, should they wish to do so, as well as improving candidate trust in such processes. Indeed, once in the workplace, autistic employees often choose not to disclose through fear of discrimination, but may wish to disclose in order to access reasonable adjustments, which should be explored directly with the candidate/employee, rather than relying on presumptions based on general autism knowledge (Romualdez et al., 2021).

Future research is required to further explore the impact on interviewer perceptions of specific behavioural and communicative differences linked with autism, in particular those that tend to be more stigmatised. Indeed, McMahon et al. (2020) found that only when ‘autistic behaviours’ were *absent* in a vignette (in particular inflexible adherence to a routine and sensory sensitivities) did disclosure have a positive impact on perceptions. Therefore, an important next step is to investigate the impact of individual behaviours of autistic and non-autistic interviewees on rater perceptions, and to assess the impact of providing autism information tailored to the candidate during diagnostic disclosure. This study also did not include measurements of demand characteristics nor social desirability bias, and such measures will be important for future research in determining which factor/s may drive positive effects of disclosure.

## Supplemental Material

sj-docx-1-aut-10.1177_13623613231203739 – Supplemental material for Disclosing an autism diagnosis improves ratings of candidate performance in employment interviews Supplemental material, sj-docx-1-aut-10.1177_13623613231203739 for Disclosing an autism diagnosis improves ratings of candidate performance in employment interviews by Jade Eloise Norris, Rachel Prosser, Anna Remington, Laura Crane and Katie Maras in Autism

## References

[bibr1-13623613231203739] Brosnan M. Mills E. (2016). The effect of diagnostic labels on the affective responses of college students towards peers with ‘Asperger’s Syndrome’ and ‘Autism Spectrum Disorder’. Autism, 20(4), 388–394. 10.1177/1362361315586721 26045542

[bibr2-13623613231203739] Crane L. Wilcock R. Maras K. Chui W. Marti-Sanchez C. Henry L. A. (2018). Mock Juror perceptions of child witnesses on the autism spectrum: The impact of providing diagnostic labels and information about autism. Journal of Autism and Developmental Disorders, 50, 1509–1519. 10.1007/s10803-018-3700-0 PMC7211190 30056502

[bibr3-13623613231203739] Flower R. L. Dickens L. M. Hedley D. (2021). Barriers to employment: Raters’ perceptions of male autistic and non-autistic candidates during a simulated job interview and the impact of diagnostic disclosure. Autism in Adulthood. 10.1089/aut.2020.0075 PMC8992918 36601643

[bibr4-13623613231203739] Gardiner E. Iarocci G. (2014). Students with autism spectrum disorder in the university context: Peer acceptance predicts intention to volunteer. Journal of Autism and Developmental Disorders, 44(5), 1008–1017. 10.1007/s10803-013-1950-4 24077739

[bibr5-13623613231203739] Gillespie-Lynch K. Brooks P. J. Someki F. Obeid R. Shane-Simpson C. Kapp S. K. Daou N. Smith D. S. (2015). Changing college students’ conceptions of autism: An online training to increase knowledge and decrease stigma. Journal of Autism and Developmental Disorders, 45(8), 2553–2566. 10.1007/s10803-015-2422-9 25796194

[bibr6-13623613231203739] Huffcutt A. I. (2011). An empirical review of the employment interview construct literature. International Journal of Selection and Assessment, 19(1), 62–81. 10.1111/j.1468-2389.2010.00535.x

[bibr7-13623613231203739] Kelley H. H. (1973). The processes of causal attribution. American Psychologist, 28(2), 107.

[bibr8-13623613231203739] Maras K. Crane L. Walker I. Memon A. (2019). Brief report: Perceived credibility of autistic witnesses and the effect of diagnostic information on credibility ratings. Research in Autism Spectrum Disorders, 68, 101442. 10.1016/j.rasd.2019.101442

[bibr9-13623613231203739] Maras K. Marshall I. Sands C. (2018). Mock Juror perceptions of credibility and culpability in an autistic defendant. Journal of Autism and Developmental Disorders, 49, 996–1010. 10.1007/s10803-018-3803-7 PMC6394789 30382444

[bibr10-13623613231203739] Maras K. Norris J. E. Nicholson J. Heasman B. Remington A. Crane L. (2020). Ameliorating the disadvantage for autistic job seekers: An initial evaluation of adapted employment interview questions. Autism, 25, 1060–1075. 10.1177/1362361320981319 33339462 PMC8108109

[bibr11-13623613231203739] McMahon C. M. Henry S. Linthicum M. (2020). Employability in autism spectrum disorder (ASD): Job candidate’s diagnostic disclosure and ASD characteristics and employer’s ASD knowledge and social desirability. Journal of Experimental Psychology: Applied, 27, 142–157. 10.1037/xap0000282 32597674

[bibr12-13623613231203739] Milton D. E. M. (2012). On the ontological status of autism: The ‘double empathy problem’. Disability & Society, 27(6), 883–887. 10.1080/09687599.2012.710008

[bibr13-13623613231203739] Morrison K. E. DeBrabander K. M. Faso D. J. Sasson N. J. (2019). Variability in first impressions of autistic adults made by neurotypical raters is driven more by characteristics of the rater than by characteristics of autistic adults. Autism, 23(7), 1817–1829. 10.1177/1362361318824104 30848682

[bibr14-13623613231203739] Norris J. E. Crane L. Maras K. (2020). Interviewing autistic adults: Adaptations to support recall in police, employment, and healthcare interviews. Autism, 24, 1506–1520. 10.1177/1362361320909174 32202435 PMC7376628

[bibr15-13623613231203739] Norris J. E. Nicholson J. Prosser R. Farrell J. Remington A. Crane L. Maras K. (under review). Perceptions of autistic and non-autistic adults in employment interviews: The role of impression management.

[bibr16-13623613231203739] Office for National Statistics. (2021). Outcomes for disabled people in the UK – Office for National Statistics. https://www.ons.gov.uk/peoplepopulationandcommunity/healthandsocialcare/disability/articles/outcomesfordisabledpeopleintheuk/2020

[bibr17-13623613231203739] Romualdez A. M. Heasman B. Walker Z. Davies J. Remington A. (2021). ‘People might understand me better’: Diagnostic disclosure experiences of autistic individuals in the workplace. Autism in Adulthood, 3, 63. 10.1089/aut.2020.0063

[bibr18-13623613231203739] Sasson N. J. Morrison K. E. (2019). First impressions of adults with autism improve with diagnostic disclosure and increased autism knowledge of peers. Autism, 23(1), 50–59. 10.1177/1362361317729526 29039208

