# Supplementary material for: Disclosing an autism diagnosis improves ratings of candidate performance in employment interviews
Source: Autism. 2023 Oct 26;28(4):1045–50. doi: 10.1177/13623613231203739 (PMC10981196; doi:10.1177/13623613231203739)
Supplement: sj-docx-1-aut-10.1177_13623613231203739 – Supplemental material for Disclosing an autism diagnosis improves ratings of candidate performance in employment interviews [file sj-docx-1-aut-10.1177_13623613231203739.docx]

# Supplementary Materials

## Supplementary Materials 1.

Diagnostic disclosure information shown to participants in the label plus information condition prior to watching and rating the mock interview (adapted from Crane et al., 2018):

**The person being interviewed has a diagnosis of autism**
**What is Autism?**

Autism is a developmental condition that affects how a person communicates with, and relates to, other people. For example, autistic people may not understand the unwritten social rules that non-autistic people inherently pick up on. Autism is diagnosed when someone has difficulties with social communication/interaction AND displays restricted interests and/or repetitive behaviours. Autistic people may also have sensory hyper- or hypo- sensitivities (e.g., over or under sensitivity to loud sounds, bright lights, etc).

**Some of the behavioural features of autism often include:**

·         **Idiosyncratic speech and odd intonations:**For example, an autistic person’s speech might be particularly flat or ‘monotone’. It may also be high-pitched, or have unusual rhythm and loudness.

·         **Literality:**autistic people can be very literal in what they say and can have difficulty understanding jokes, metaphor, and sarcasm. For example, “that’s cool” might be taken to mean that it is cold.

·         **Facial expressions and gestures:**autistic people may use unusual, or a limited range of, facial expressions. They can find it difficult to use expressive gestures appropriately and to convey the meaning of what they are saying.

·         **Reading interpersonal cues:**autistic people can find it difficult to interpret and respond appropriately to subtle social cues, such as adapting their responses based on the facial expressions, tone of voice, and body language of the person they are talking to.

·         **Topics of conversation:**autistic people sometimes go off-topic in their story telling, and find it difficult to tell their story according to the listener’s needs.

·         **Answering questions:**autistic people may provide non-specific answers which are less focused or clear, and may use fewer examples in their answers.

·         **Repetitive, nervous, and ‘stimming’ behaviours:**autistic people often show unusual movements, which might include rocking, hand flapping, finger flicking, hand wringing, playing with hair or clothing, leg movements, nail biting, twitchy, and repetitive movements.

·        **Inappropriate eye contact:**autistic people sometimes make unusual eye contact, or avoid making eye contact altogether.

Importantly, **autism is a spectrum condition**. This means that, while autistic people share certain difficulties, they are affected by it in different ways and not all autistic people will display the behaviours just described, or to the same degree. It is often referred to as a ‘hidden’ disability, because it is not always obvious that a person has an autism diagnosis.
